# The Resistome of Low-Impacted Marine Environments Is Composed by Distant Metallo-β-Lactamases Homologs

**DOI:** 10.3389/fmicb.2018.00677

**Published:** 2018-04-05

**Authors:** Erica L. Fonseca, Bruno G. N. Andrade, Ana C. P. Vicente

**Affiliations:** Laboratório de Genética Molecular de Microrganismos, Instituto Oswaldo Cruz, Fundação Oswaldo Cruz, Rio de Janeiro, Brazil

**Keywords:** resistome, metallo-β-lactamases, marine environment, VIM, SPM-1, distant homolog, pristine environment, antibiotic resistance gene

## Abstract

The worldwide dispersion and sudden emergence of new antibiotic resistance genes (ARGs) determined the need in uncovering which environment participate most as their source and reservoir. ARGs closely related to those currently found in human pathogens occur in the resistome of anthropogenic impacted environments. However, the role of pristine environment as the origin and source of ARGs remains underexplored and controversy, particularly, the marine environments represented by the oceans. Here, due to the ocean nature, we hypothesized that the resistome of this pristine/low-impacted marine environment is represented by distant ARG homologs. To test this hypothesis we performed an *in silico* analysis on the Global Ocean Sampling (GOS) metagenomic project dataset focusing on the metallo-β-lactamases (MβLs) as the ARG model. MβLs have been a challenge to public health, since they hydrolyze the carbapenems, one of the last therapeutic choice in clinics. Using Hidden Markov Model (HMM) profiles, we were successful in identifying a high diversity of distant MβL homologs, related to the B1, B2, and B3 subclasses. The majority of them were distributed across the Atlantic, Indian, and Pacific Oceans being related to the chromosomally encoded MβL GOB present in *Elizabethkingia* genus. It was observed only a reduced number of metagenomic sequence homologs related to the acquired MβL enzymes (VIM, SPM-1, and AIM-1) that currently have impact in clinics. Therefore, low antibiotic impacted marine environment, as the ocean, are unlikely the source of ARGs that have been causing enormous threat to the public health.

## Introduction

Antibiotic resistance genes (ARGs) represent a significant burden to public health and economies, since it directly affects the treatment and management of infectious diseases. Microbial resistance to natural and synthetic antibiotics has been arising after their introduction in the clinical settings, suggesting the pre-existence of natural reservoirs of antibiotic resistance determinants, which is called environmental resistome. The resistome consists of all resistance genes from pathogenic, non-pathogenic or antibiotic-producing microbiota that are direct or indirectly involved with antimicrobial resistance (Wright, 2007; Forsberg et al., 2012).

In general, the natural environment, particularly the soil, has been pointed as the origin and reservoir of ARGs that further emerge in clinical settings (D’Costa et al., 2011). In fact, many of the antimicrobial compounds have been isolated from soil microorganisms and, in such niche, antibiotic producers and non-producers have developed self-protection mechanisms in response to the presence of these naturally produced antibiotics. These mechanisms have been evolving and fixing in such environmental microbiota (representing its resistome), favoring the presence and maintenance of antibiotic resistance in soil environments (Nesme and Simonet, 2015). Similarly to soil, aquatic environments, particularly those under the influence of anthropogenic interventions, such as wastewater plants, hospital sewage and aquaculture farms, also present a resistome mainly composed by ARGs identical or very similar to those found in clinics (Baquero et al., 2008; Figueira et al., 2011; Pruden et al., 2012; Tacão et al., 2012; Fróes et al., 2016). On the other hand, studies have demonstrated that the resistome of minimally impacted environments was composed by a higher proportion of distant homologs related to intrinsic and constitutive resistance determinants instead of horizontally acquired ARGs currently circulating in clinical pathogens (Storteboom et al., 2010; Chen et al., 2013; Hatosy and Martiny, 2015).

The oceans cover more than 70% of the Earth’s surface and hold a huge under-explored microbial community (Versluis et al., 2015; Fitzpatrick and Walsh, 2016). Its dynamics and metabolic diversity suggest that, even pristine and low-impacted environments as oceans, could play an unpredicted role in the natural history of antibiotic resistance. However, this issue remains underexplored and controversy (Thaller et al., 2010; Ushida et al., 2010; Hatosy and Martiny, 2015).

In fact, the origin of ARGs is far from be understood, and one example is the metallo-β-lactamase (MβL) coding genes. Although some MβLs had their origin in environmental bacteria from soil (Naas et al., 2003), these enzymes remain continuously emerging in pathogens with no clue about their environmental sources and reservoirs, as found for VIM and SPM-1, among others (Wachino et al., 2011; Pollini et al., 2013; Gudeta et al., 2016; Berglund et al., 2017). The MβLs belong to a highly diverse and ancient family of enzymes that hydrolyze carbapenems, the last therapeutic choice for treating human infections caused by Gram-negative pathogens (Hall et al., 2004). These enzymes exhibit structural and functional heterogeneity, sharing conservation only in specific domains within the catalytic site, such as the zinc-binding motif HxHxD. Based on the nature of the residues coordinating the metal ions, these enzymes are classified into three distinct subclasses (B1, B2, and B3) (Bebrone, 2007; Palzkill, 2013). The subclass B1 enzymes, like VIM, SPM-1, and IMP, are clinically relevant and frequently encoded in mobile genetic elements (Walsh et al., 2005; Fonseca et al., 2015), which accounts for their spread among pathogens. On the other hand, most of subclasses B2 and B3 members are encoded in the chromosome of environmental bacteria. The VIM enzymes have been reported in clinical Gram-negative bacteria from several genera worldwide (Walsh et al., 2005), while the *bla*_SPM-1_ was found, so far, in a *Pseudomonas aeruginosa* lineage prevalent and spread in Brazil, in association with an Integrative and Conjugative Element (ICE) (Fonseca et al., 2010, 2015). However, some B1 MβLs are found in environmental bacteria, such as JOHN-1 that is intrinsically encoded in the chromosome of the fish pathogen *Flavobacterium johnsoniae*, an environmental bacteria ubiquitous in soil and fresh water environments (Naas et al., 2003).

In the subclass B2, the CphA is the most studied enzyme and presents a very narrow and specific substrate profile compared with other class B enzymes (Segatore et al., 1993). It is intrinsically encoded in the chromosome of *Aeromonas* species, which are ubiquitous in water environments and often emerge in clinics (Nikiforov et al., 2014). The subclass B3 includes some clinically relevant MβL enzymes, such as L1, FEZ-1, AIM-1, and GOB (Horsfall et al., 2011). The GOB is part of the core genome of all recognized *Elizabethkingia* species, revealing a long evolutionary history. In fact, bacteria from this genus are widely distributed in nature, including fresh and salt water, and its association with human infections has already been reported (Issack and Neetoo, 2011). The GOB-type enzymes are characterized by the presence of a glutamine residue at position 116 in the conserved zinc-binding motif, differing from all other members of subclass B3 and B1, which have a histidine at this position (Bellais et al., 2000; Galleni et al., 2001). The L1 enzyme (Crowder et al., 1998) is found in the chromosome of *Stenotrophomonas maltophilia*, which is a ubiquitous bacterium with an opportunistic potential, often associated with severe lung infection in newborn children and immune-compromised patients (Çıkman et al., 2016). Finally, the AIM-1 was found in a *P. aeruginosa* clinical strain and, different from all subclass B3, its coding gene was flanked by two ISCR elements, which would favor its mobilization (Yong et al., 2012).

Considering that the soil and impacted aquatic environments have a high density of ARGs similar to those found in clinics, probably due to the antibiotic presence, we hypothesized here that the oceans, a dynamic biome devoid of a direct antibiotic selective pressure, would harbor a resistome represented by distant ARG homologs. Thus, in order to test our hypothesis, we focused our study on the search for MβLs due to their current public health relevance and to the lack of information concerning their origin and natural reservoirs. For this purpose, the Global Ocean Sampling (GOS) metagenome project, which contains a huge and diverse dataset considering the marine environment, was analyzed using an *in silico* approach based on HMM profiles. It was revealed a high diversity of distant MβL homologs spread in oceans. Therefore, this study was original in showing evidences that a low-impacted/pristine aquatic environment, such as oceans, are unlikely the source of MβLs currently emerging in clinics.

## Materials and Methods

### Datasets

The GOS project includes a set of DNA sequences recovered from 84 seawater sample sites, including a variety of marine environments, which were collected during a global circumnavigation expedition from 2003 to 2006 (Yooseph et al., 2007). Therefore, it provides a huge amount of information of several marine environments with no or low anthropogenic influence. This large dataset allowed not only the investigation of the microbial diversity within the marine environment, but also the study of their genetic composition in terms of, for example, antimicrobial resistance and virulence. Thus, in order to access the marine resistome, the nucleotide sequence of the GOS and Whale Fall mat metagenomes were retrieved from the *Community Cyberinfrastructure for Advanced Microbial Ecology Research and Analysis* (CAMERA) database (Sun et al., 2011), which is now assessable on the iMicrobe Project^1^.

### Screening for Metallo-β-Lactamases (MβLs) Homologs

The marine MβL homologs were predicted using a computational method based on Hidden Markov Model (HMM) profiles built with the HMMer v3 software (Eddy, 2009). Representative curated MβL sequences from each subclass were retrieved from the Universal Protein Knowledgebase (UniProtKB) (Hinz and UniProt Consortium, 2010), grouped according to their subclasses, and aligned with the Mafft v5 software using the default settings (Katoh et al., 2005). Three HMM profiles were built with the resulting alignments relative of each subclass, using the “hmmbuild” algorithm as the default settings. The metagenomics nucleotide sequences were translated in the six frames using the Tranqseq software included in the European Molecular Biology Open Source Suit (EMBOSS). The method first performed a quality control of the metagenomics reads and then searched each fragment using the HMM profiles as probes with an e-value cutoff of 10^-20^. To find the closest MβL homolog, each sequence was queried against the NCBI non-redundant protein database (NR) using the BLASTp+ software with the same similarity threshold of the HMMer search (10^-20^). Sequences with approximately 40% amino acid identity and 70% amino acid sequence coverage to their curated homologs were considered. These parameters provide a relatively secure functional inference based on empirical evidence (Tian and Skolnick, 2003). The resulted sequences were visually screened for MβL conserved zinc-binding motifs, such as the H116 × H118 × D120 that is part of the catalytic site shared by all MβL enzymes (Hall et al., 2004).

### Phylogenetic Analyses

In order to stablish the relationship between the predicted environmental MβL homologs and their curated functional counterparts, a phylogenetic reconstruction was performed with the maximum likelihood method and WAG+I+G amino acid substitution model in the MEGA6 software (Tamura et al., 2011). Confidence values of nodes were estimated using 1,000 bootstrap replications. *Sulfolobus solfataricus* MβL was used as the outgroup for subclass B1/B2, and *Pyrococcus horikoshii* MβL was used as the outgroup for subclass B3, as described elsewhere (Hall et al., 2004). As observed in **Table 1**, identical metagenomics sequences were recovered from same sites, and in this case, only one representative sequence was included in the phylogenetic analysis.

**Table 1:** Global Ocean Sampling (GOS) marine sites and the metallo-β-lactamase distant homologs.

Site	Number of reads	Location	Subclass	Homolog to	Amino acid identity (%)	Amino acid similarity (%)	Protein coverage (%)
GS048a	2	French Polynesia	B1	VIM	55	71	65
GS049	1	French Polynesia			53	73	78
GS032	2	Galapagos		SPM-1	53	71	91
GS020	7	Panama		JOHN-1	56	74	90
GS000a	1	Sargasso Sea	B2	CphA	92	94	70
GS000d	3	Sargasso Sea	B3		41	63	91
GS001c	1	Sargasso Sea			40	57	86
GS015	2	Caribe			42	60	91
GS018	3	Caribe			40	56	88
GS035	1	Galapagos			38	56	91
GS031	3	Galapagos		GOB	39	57	87
GS029	1	Galapagos			39	57	98
GS108a	1	Indian Ocean			40	61	76
GS119	1	Indian Ocean			44	61	85
GS113	1	Indian Ocean			40	59	82
GS047	1	French Polynesia			40	58	83
GS000a	1	Sargasso sea		AIM-1	52	73	86
GS029	5	Galapagos		L1	85	92	99
Whale fall mat	4	United States		BJP-1	46	61	88

## Results and Discussion

The emergence of ARGs in clinics, especially those conferring resistance to last resource antibiotics such as carbapenems, has been raising questions concerning their reservoir, origin and evolution (Cornaglia et al., 2011; Gudeta et al., 2016). In this way, the search for these ARGs in minimally impacted environments would be pivotal for understanding the emergence of the modern antibiotic resistance (Chen et al., 2016). However, the majority of studies on environmental resistome has been exploring microbiomes from environments with anthropogenic impact, which may impose a bias toward the recovery of ARGs very similar to those found in clinics (Allen et al., 2010).

**FIGURE 1:**
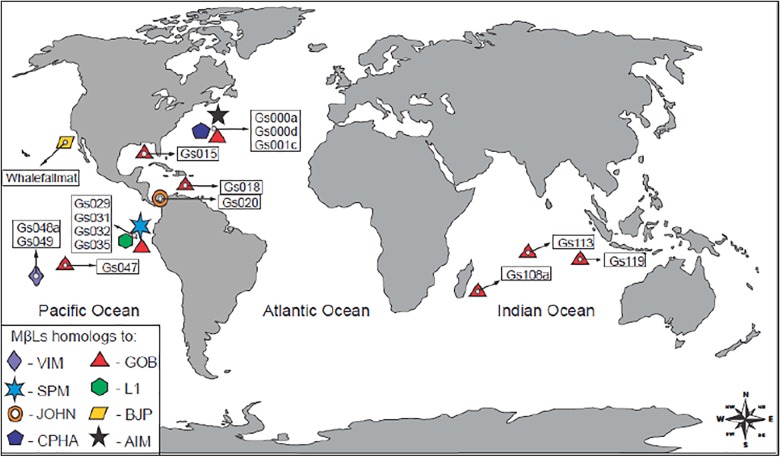
Geographical distribution of MβLs homologs in the oceans. The corresponding sites are highlighted in boxes.

Here, we performed metagenomics analyses for mining MβLs in a dataset represented by low-impacted marine environments. We applied HMM profiles, which would allow the recognition of closely related, as well as distant MβL homologs. In this way, we identified a high diversity of distant MβL homologs related to the three subclasses across 16/84 GOS marine sites, corresponding to superficial water (0.1–12 m) from coastal areas, open oceans, coral reef, coastal upwelling, abyssopelagic zone, mangrove and lake (**Figure 1** and **Table 1**). It was identified 41 metagenomics sequences (**Table 1**) presenting all the conserved motifs that characterize the MβLs (**Supplementary Figures S1**–**S3**).

**FIGURE 2:**
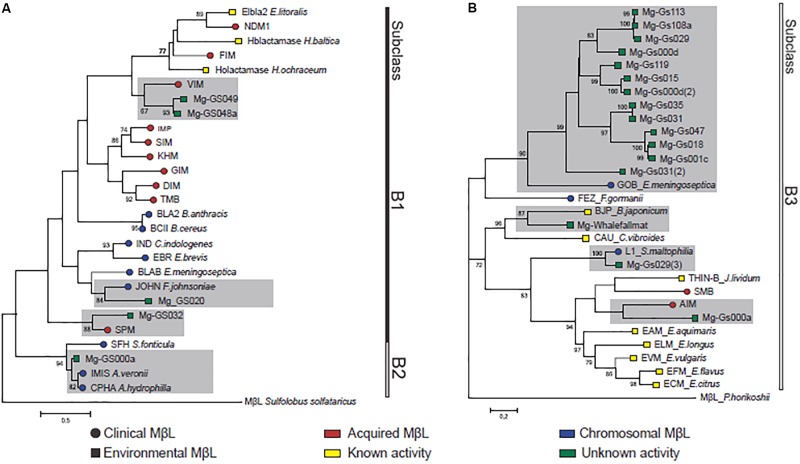
Phylogenetic reconstruction showing the evolutionary relationships between the MβL homologs predicted in this study and representative sequences of curated acquired and chromosomally encoded MβLs from subclass B1/B2 **(A)** and subclass B3 **(B)**. Clusters with metagenomic sequences identified as MβL homologs are highlighted in gray.

The phylogenetic reconstruction revealed that the metagenomics sequences clustered with curated MβLs from the three subclasses (**Figure 2**). Three subclusters were defined within the major cluster corresponding to subclass B1, indicating a relationship of four metagenomics sequences with the MβLs VIM, SPM-1, and JOHN-1. One unique sequence clustered with the subclass B2, represented by the CphA enzyme. The remaining 28 sequences belonged to subclass B3, and they were distributed in four subclusters represented by the curated GOB, AIM-1, BJP-1, and L1 enzymes (**Figure 2**). Interestingly, the majority of the sequences showed identity with chromosomally encoded MβLs (CphA, JOHN-1, L1, BJP-1, and GOB), while few were related with the horizontally acquired VIM, SPM-1 and AIM-1 MβL enzymes (**Figure 2**). It is worth note that such chromosomally encoded enzymes do not represent a threat to antibiotic resistance emergence in clinics since, at first, they are not submitted to horizontal gene transfer. Moreover, those sequences related to acquired MβL enzymes would not represent a direct a link to resistance emergence, since they are distant homologs, corroborating our hypothesis. Even though, some of these homologs were related to enzymes with clinical relevance, such as VIM, SPM-1, AIM-1, GOB, L1, and CphA.

Sequences recovered from distinct GOS sites presented amino acid identity with the B1 horizontally acquired VIM (55%) and SPM-1 (53%), which are prevalent among pathogenic and opportunistic bacteria (**Table 1**, **Figure 1**, and **Supplementary Figure S1**). The VIM homologs (*n* = 3) were found in the superficial water (1 m of depth) of different sites (Gs048a and Gs049) next to Cooks bay, French Polynesia, placed ∼8,000 km apart from the South America West Coast. The SPM-1 homologs (*n* = 2) were recovered from the superficial water of a mangrove on Isabella Island (site Gs32), Galapagos archipelago, 1,000 km apart from the South America West Coast (**Figure 1**). On the other hand, we also found metagenomics sequences in a fresh water lake (site Gs020) of the Panama Channel that shared 56% amino acid identity with the B1 MβL JOHN-1 (*n* = 7) (**Figure 1** and **Supplementary Figure S1**), which is encoded in the chromosome of *F*. *johnsoniae*.

The subclass B2 homolog identified presented a remarkable amino acid identity (91%) with the CphA enzyme (*n* = 1) (**Table 1** and **Supplementary Figure S1**). As found for all functional B2 MβLs, this metagenomics sequence contained the H116N amino acid substitution in its catalytic site. This sequence was recovered from an oceanic pelagic zone in the Sargasso sea (Gs000a site), a site located more than 7,000 km apart from the United States East coast and, therefore, supposed to have low anthropogenic influence. Thus, the high amino acid identity observed between this metagenomics sequence and CphA, and the intrinsic association of this MβL with *Aeromonas* species, which are ubiquitous in aquatic environments, would indicate the presence of this bacterial genus in such pristine marine environment.

Subclass B3 distant homologs were the most prevalent and widely distributed metagenomic sequences, in particular, those related to the GOB MβL (*n* = 18) (**Table 1** and **Figure 1**). They shared 38–44% amino acid identity with GOB-1 (**Table 1**, **Figure 2**, and **Supplementary Figure S2**), and were distributed in four major clusters, apart from the canonical sequence harbored by clinical *E. meningosepticum* (**Figure 2**). Interestingly, these clusters were not niche specific since the GOB homologs within the same cluster were recovered in distinct sites corresponding to different marine environments. All GOB homologs presented the G116 amino acid residue in the catalytic site, which characterized the GOB enzymes (**Supplementary Figure S2**) (Horsfall et al., 2011). These sequences were recovered from 11 sites spatially distributed in both coastal and open ocean waters of the Atlantic (Sargasso and Caribbean seas), Pacific (the Galapagos Islands and French Polynesia) and Indian oceans (**Table 1** and **Figure 1**). Noteworthy, is the presence of same GOB sequences in distinct sites (Gs018/Gs001c, Gs113/108a, and Gs031/Gs035). Considering that GOB is a chromosomally encoded enzyme, this finding is an evidence of a wide distribution of GOB hosts through these environments. On the other hand, a diversity of GOB sequences were observed in the same sites (Gs031 and Gs000d) (**Table 1** and **Figures 1**, **2**). Once there is no selective pressure imposed by antibiotics in these sites, the widespread presence and distribution of GOB indicate that these enzymes could play other roles in the bacterial physiology. Other subclass B3 MβLs were identified in minor proportion (**Table 1**). One metagenomics sequence sharing 52% amino acid identity with the B3 subclass AIM-1 was found in the Sargasso Sea (site Gs000a) (**Table 1**, **Figures 1**, **2**, and **Supplementary Figure S3**). Homolog sequences (*n* = 5) related with the B3 L1 MβL (88% amino acid identity) was found in an upwelling region near the Fernandina Island, in the Galapagos archipelago (site Gs029) (**Table 1**, **Figures 1**, **2**, and **Supplementary Figure S3**). Finally, homolog sequences (*n* = 4) presenting 46% amino acid identity with BJP-1, which is chromosomally encoded in the nitrogen-fixing *Bradyrhizobium japonicum* (Stoczko et al., 2006), was identified in the Whale Fall metagenomic project (site Whalefallmat) in the Pacific Ocean (**Table 1**, **Figures 1**, **2**, and **Supplementary Figure S3**). All these sites are considered to be devoid of antibiotic pressure, both due to the low anthropogenic influence and to the planktonic nature of the bacteria sampled.

All these analyses are consistent with our hypothesis that the ocean’s resistome is represented by distant MβL homologs and not by MβLs that have been emerging in clinics. In fact, the under-representation of homologs related with several others MβLs of clinical importance, together with the identification of only three homolog sequences related with horizontally acquired MβLs, indicate that the recovery of ARGs similar to those found in clinics in pristine environments, such as oceans, is more likely to represent a vestige of anthropogenic impact than an evidence of ARG source/reservoirs (Thaller et al., 2010; Ushida et al., 2010). The marine resistome profile found for MβLs in oceanic regions with low anthropogenic influence revealed here seems to be a general feature. For example, a recent study mining other class of antibiotic hydrolyzing enzymes, the serine-β-lactamases, revealed a similar scenario in pristine/low-impacted environments, in which only distant homologs were identified in oceans (Fróes et al., 2016).

Therefore, our study provides original evidences that the oceans are unlikely the source of the ARGs emerging in clinical pathogens. In this way, environments under high antibiotic selective pressure, whatever as a consequence of its natural production or due to human use, have to be considered the frontier to monitor the ARG emergence that have been compromising the infectious diseases treatment.

## Author Contributions

EF: participated in the interpretation and discussion of the results. Wrote the manuscript and revised it critically for important intellectual content. Participated in the final approval of the version to be published. BA: participated in the study conception, performed the *in silico* analyses, and contributed to acquisition and data analysis. AV: involved in all steps, which included the study design and conception, execution supervision, analysis, interpretation and discussion of the results; wrote the manuscript and revised it critically for important intellectual content and participated in the final approval of the version to be published.

## Conflict of Interest Statement

The authors declare that the research was conducted in the absence of any commercial or financial relationships that could be construed as a potential conflict of interest.

## Footnotes

1 http://data.imicrobe.us

## Supplementary Material

FIGURE S1 Alignments of curated subclass B1 and B2 metallo-β-lactamases and their environmental homologs. Identical residues and conserved substitutions are highlighted in gray, residues from the catalytic site are highlighted in green.

FIGURE S2 Alignment of the curated subclass B3 GOB-1 enzyme and its environmental homologs. Identical residues and conserved substitutions are highlighted in gray, residues from the catalytic site are highlighted in green.

FIGURE S3 Alignments of curated subclass B3 MβLs and their environmental homologs. Identical residues and conserved substitutions are highlighted in gray, residues from the catalytic site are highlighted in green.
