# Supplementary figures and images for: The Resistome of Low-Impacted Marine Environments Is Composed by Distant Metallo-β-Lactamases Homologs
Source: Front Microbiol. 2018 Apr 5;9:677. doi: 10.3389/fmicb.2018.00677 (PMC5895761; doi:10.3389/fmicb.2018.00677)

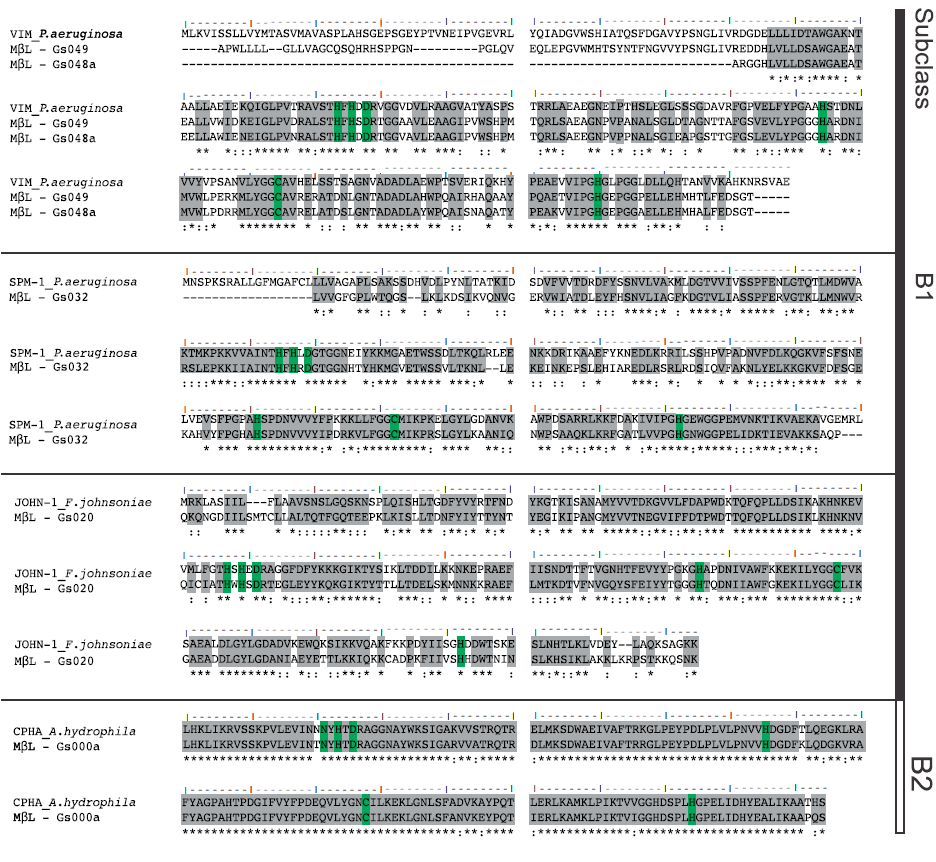

Supplement: FIGURE S1 — Alignments of curated subclass B1 and B2 metallo-β-lactamases and their environmental homologs. Identical residues and conserved substitutions are highlighted in gray, residues from the catalytic site are highlighted in green. [file Image_1.TIF]

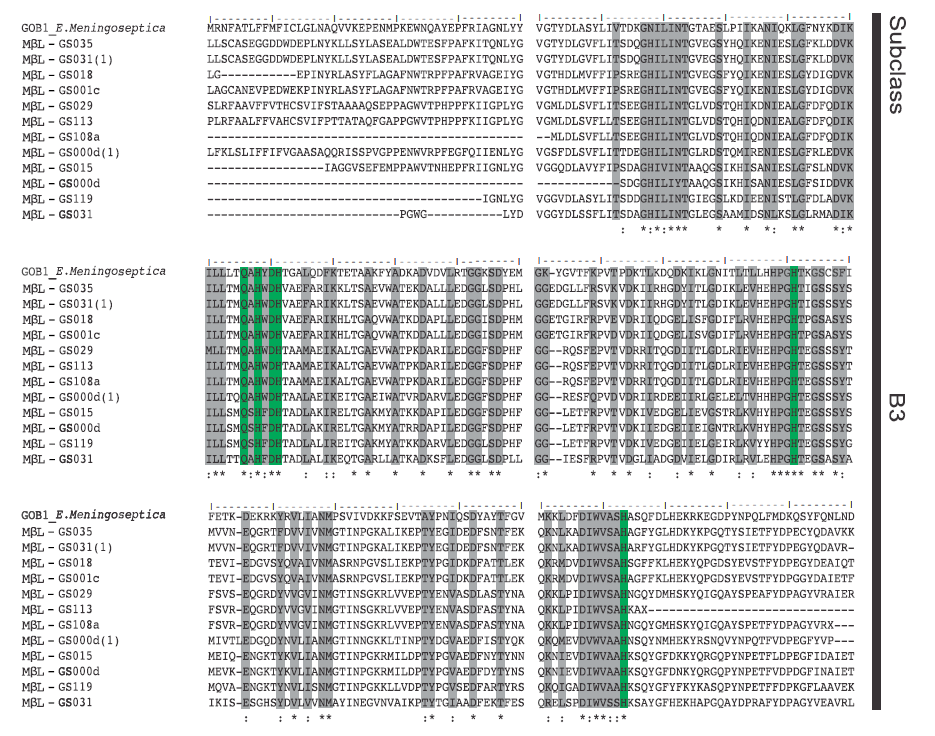

Supplement: FIGURE S2 — Alignment of the curated subclass B3 GOB-1 enzyme and its environmental homologs. Identical residues and conserved substitutions are highlighted in gray, residues from the catalytic site are highlighted in green. [file Image_2.TIF]

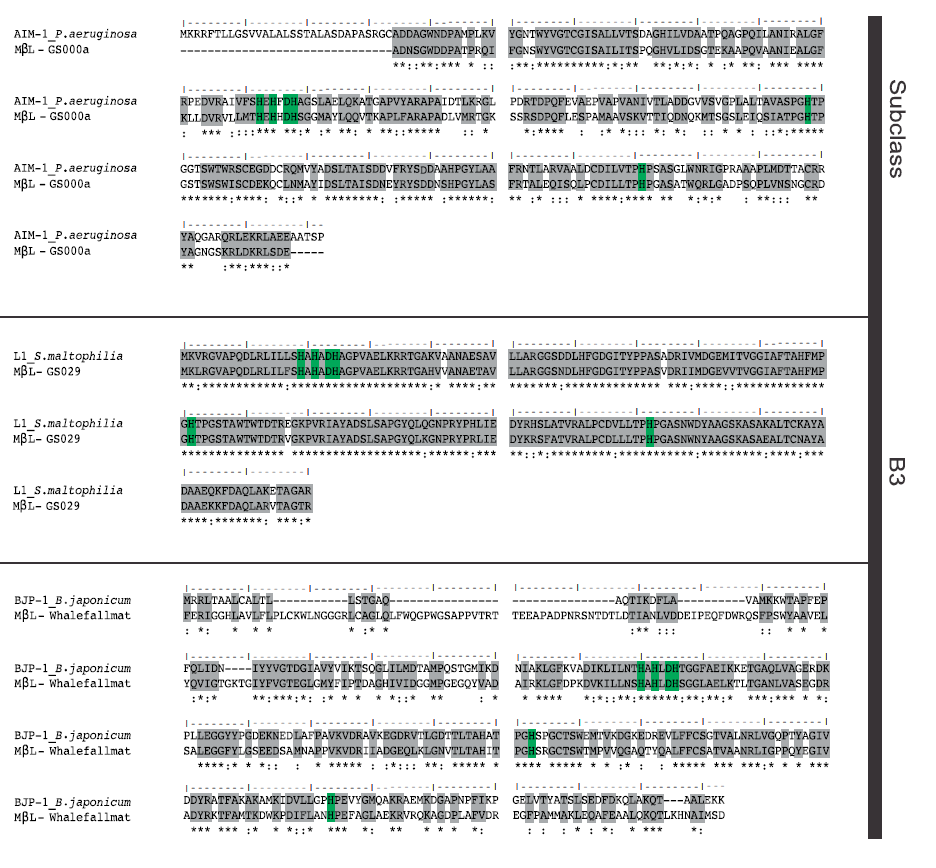

Supplement: FIGURE S3 — Alignments of curated subclass B3 MβLs and their environmental homologs. Identical residues and conserved substitutions are highlighted in gray, residues from the catalytic site are highlighted in green. [file Image_3.TIF]
